# Atony and Associated Pathological Conditions of the Rectum and Colon; with Mechanical Methods of Treatment

**Published:** 1905-10

**Authors:** 


### Atony and Associated Pathological Conditions of the Rectum and Colon; with Mechanical Methods of Treatment.

Fenton B. Turck advises the use of massage and stimulation of the atonic intestine by the use of small rubber bags inserted in the rectum and sigmoid flexure, and inflated with air. The inflation can be used steadily for the desired amount of time, or the bag may be alternately relaxed and inflated again, thus producing a kind of massage of the intestine. Atony of the intestine is the result of fatigue of toxins generated by the intestine, and antitoxins may be also generated, which will restore the intestine to its normal condition. The toxins of fatigue are not dializable, and remain where they are formed. Massage hastens the union of antitoxins with toxins. The abdominal circulation is also an important factor in atony of the intestine, and massage by inflation stimulates the circulation. Drugs, surgery, general gymnastics, and the various mechanical methods of treatment have all failed in curing atony. The injection of air confined in the rubber bag places the amount of distention to be used under the operator's control. It may be made intermittent. Hemorrhoids, ulcers, proctitis, all are benefited, as well as prolapse of the bowel, and various associated uterine conditions. The results of the author's experience have shown that the restoration of function in the intestine is permanent.—*Medical Record*, October 7, 1905.

